# Nomogram risk prediction model for acute respiratory distress syndrome following acute kidney injury

**DOI:** 10.3389/fmed.2025.1563425

**Published:** 2025-04-09

**Authors:** Hui Lin, Yilin Ren, Jing Cui, Junnan Guo, Mengzhu Wang, Lihua Wang, Xiaole Su, Xi Qiao

**Affiliations:** ^1^Department of Nephrology, Second Hospital of Shanxi Medical University, Taiyuan, China; ^2^Shanxi Kidney Disease Institute, Taiyuan, China; ^3^Kidney Research Center of Shanxi Medical University, Taiyuan, China; ^4^Department of Endocrinology, Air Force Medical Center, Beijing, China

**Keywords:** acute respiratory distress syndrome (ARDS), acute kidney injury (AKI), risk factor, early prediction, nomogram

## Abstract

**Background:**

Acute respiratory distress syndrome (ARDS), a severe form of respiratory failure, can be precipitated by acute kidney injury (AKI), leading to a significant increase in mortality among affected patients. This study aimed to identify the risk factors for ARDS and construct a predictive nomogram.

**Methods:**

We conducted a retrospective analysis of 1,241 AKI patients admitted to the Second Hospital of Shanxi Medical University from August 25, 2016, to December 31, 2023. The patients were divided into a study cohort (1,012 cases, including 108 with ARDS) and a validation cohort (229 cases, including 23 with ARDS). Logistic regression analysis was employed to identify the risk factors for ARDS, which were subsequently incorporated into the development of a nomogram. The predictive performance of the nomogram was assessed by AUC, calibration plots, and decision curve analyses, with external validation also performed.

**Results:**

Six risk factors were identified and included in the nomogram: older age (OR = 1.020; 95%CI = 1.005–1.036), smoking history (OR = 1.416; 95%CI = 1.213–1.811), history of diabetes mellitus (OR = 1.449; 95%CI = 1.202–1.797), mean arterial pressure (MAP; OR = 1.165; 95%CI = 1.132–1.199), higher serum uric acid levels (OR = 1.002; 95%CI = 1.001–1.004), and higher AKI stage [(stage 1: reference), (stage 2: OR = 11.863; 95%CI = 4.850–29.014), (stage 3: OR = 41.398; 95%CI = 30.840–52.731)]. The AUC values were 0.951 in the study cohort and 0.959 in the validation cohort. Calibration and decision curve analyses confirmed the accuracy and clinical utility of the nomogram.

**Conclusion:**

The nomogram, which integrates age, smoking history, diabetes mellitus history, MAP, and AKI stage, predicts the risk of ARDS in patients with AKI. This tool may aid in early detection and facilitate clinical decision-making.

## Introduction

Acute kidney injury (AKI) is one of the most prevalent forms kidney injury and has garnered significant attention as a major public health concern (1). It affects 10–15% of hospitalized patients, with an overall mortality rate of approximately 21%, including a striking 40–60% mortality rate among critically ill patients, and this figure continues to rise (2, 3). An growing body of evidence suggests that the high morbidity and mortality associated with AKI are at least partially attributable to subsequent secondary multiorgan failure (4). Pulmonary dysfunction is likely one of the most prominent clinical manifestations of AKI and has been extensively investigated as a distant organ effect (5). Respiratory failure is frequently observed in patients with AKI and can progress to acute lung injury, ultimately leading to acute respiratory distress syndrome (ARDS), with mortality rates escalating to 60–80% (6).

A single-center retrospective cohort study indicates that the coexistence of AKI and ARDS is associated with worse outcomes compared to patients with AKI or ARDS alone. Among patients diagnosed with both conditions, those who developed ARDS subsequent to AKI exhibited the highest mortality rate (7). AKI directly results in decreased urine output, leading to fluid overload and pulmonary edema. This has traditionally been regarded as the primary cause of pulmonary dysfunction in patients with AKI (8). Furthermore, the lungs possess an extensive capillary network (9). Chemical mediators such as pro-inflammatory uremic toxins can readily compromise the integrity of the alveolar-capillary membrane. This compromise can also lead to lung injury and dysfunction. Moreover, this condition cannot be adequately addressed simply by treating uremia and controlling fluid balance through dialysis (8). Consequently, ARDS after AKI (10) is associated with a higher mortality rate. Early diagnosis and treatment of ARDS occurrence following AKI are therefore of paramount importance.

However, the lack of risk nomograms for ARDS occurrence following AKI has rendered the early and accurate diagnosis of ARDS in AKI patients a persistent challenging in clinical practice. We aimed to construct a prediction model for ARDS occurrence following AKI that integrates multiple risk factors. This model would facilitate the early identification of high-risk AKI patients, enabling the prompt implementation of appropriate preventive and intervention measures, thereby effectively reducing the incidence and mortality rate of ARDS after AKI.

## Methods

### Design and study cohort

From August 25, 2016, to December 31, 2023, we conducted a retrospective data collection of consecutively hospitalized patients were retrospectively collected from the Department of Nephrology, Second Hospital of Shanxi Medical University. Given the relatively high misdiagnosis rate of AKI as reported by Yang et al. (11), we employed a comprehensive screening approach. We identified AKI patients using both the 2012 Kidney Disease: Improving Global Outcomes (KDIGO) guidelines (12) and the extended diagnostic criteria proposed by Yang et al. (11). Furthermore, we utilized the International Classification of Diseases, 10th Revision, Clinical Modification (ICD-10-CM) codes to capture reported AKI cases. The specific codes included N17.0 (Acute kidney failure with tubular necrosis), N17.1 (Acute kidney failure with acute cortical necrosis), N17.2 (Acute kidney failure with medullary necrosis), N17.8 (Other acute kidney failure), and N17.9 (Acute kidney failure, unspecified) (13). This single-center retrospective cohort study was designed to investigate risk factors associated with the development of ARDS in AKI patients. The study strictly adheres to the Strengthening the Reporting of Observational Studies in Epidemiology (STROBE) guidelines (Supplementary Table S1) (14).

### Inclusion criteria

Initially, utilizing the electronic medical record system, we selected the medical records of all patients who were admitted for the first time during the study period, spanning from the start to the end date. Next, we excluded patients who had fewer than two serum creatinine (SCr) tests conducted during their hospital stay. AKI, as defined by KDIGO guidelines, served as our primary screening criterion: [an increase in SCr by ≥0.3 mg/dL (≥26.5 μmol/L) within 48 h; or an increase in SCr to ≥1.5 times the baseline, which is known or presumed to have occurred within the past 7 days] (11). We reconfirmed the diagnosis of AKI by rechecking the changes in SCr via the laboratory information system. For patients with SCr measurements taken more than 7 days apart and for those who had recovered from AKI, we extended our screening criteria: [an increase or decrease in SCr of at least 26.5 μmol/L during hospitalization; or an increase or decrease in SCr of at least 50% (relative to baseline SCr levels)] as supplementary indicators of AKI (11).

### Exclusion criteria

1. Patients who had progressed to end-stage kidney disease (ESKD), initiated kidney replacement therapy (KRT), undergone kidney transplantation, or had a peak SCr level below 53 μmol/L upon admission.
2. Patients experiencing severe pulmonary events at the time of AKI diagnosis, including but not limited to: chronic obstructive pulmonary disease (COPD), interstitial lung diseases (such as idiopathic pulmonary fibrosis, sarcoidosis, hypersensitivity pneumonitis, etc.), pulmonary arterial hypertension (PAH), asthma (particularly poorly controlled or severe cases), bronchiectasis, cystic fibrosis, lung trauma or pulmonary dysfunction due to post-tuberculosis sequelae, lung cancer with respiratory dysfunction, and cardiogenic pulmonary edema caused by heart failure.
3. Patients under 18 years of age or pregnant/lactating females.
4. Patients with a hospital stay of fewer than 3 days.
5. Patients whose changes in SCr met identification criteria but could not be attributed to AKI (e.g., a decrease in SCr following amputation).

Ultimately, confirmed AKI patients were divided into a study cohort and a validation cohort. This study was approved by the Medical Ethics Committee of the Second Hospital of Shanxi Medical University (Approval No.: 2023-YX-173).

### Definition of ARDS

According to the Berlin definition (15), ARDS is defined as an acute and severe respiratory failure resulting from extensive damage to the alveolar-capillary membrane. Symptoms typically manifest acutely within 48 h following the inciting event. Chest imaging, such as X-ray or CT scan, demonstrates bilateral pulmonary infiltrates that cannot be fully attributed to other causes, including but not limited to heart failure, severe anemia, tumor-related pulmonary infiltrates, or lymphangitic carcinomatosis. The severity of hypoxemia in ARDS is categorized based on the PaO₂/FiO₂ ratio: mild ARDS is defined by a ratio of 200–300, moderate ARDS by a ratio of 100–200, and severe ARDS by a ratio <100. Oxygenation assessment should be conducted der a condition of positive end-expiratory pressure (PEEP) ≥ 5 cmH₂O.

### Collection of clinical variables

Data were retrospectively collected in a consecutive manner. Baseline clinical and demographic information was gathered at the time of admission during the initial testing. Categorical variables included sex (male, female), smoking history (no, yes), alcohol consumption (no, yes), diabetes mellitus history (no, yes), AKI stage (stage 1, 2, 3), etiology of AKI (pre-renal, intrinsic, postrenal, unclassified), and specific injury factors of AKI, such as decompensated cirrhosis (DCC), renal artery and occlusion (RAS and RAO), hemorrhage, acute gastroenteritis (AGE), nephrotoxic drugs/toxins, contrast-induced nephropathy (CIN), sepsis, surgery, rhabdomyolysis, urinary tract calculi or stenosis, prostatism, urologic/pelvic cancer, and neurogenic bladder (11, 16). Continuous variables encompassed age (years), body mass index (BMI, kg/m^2^), albumin (ALB, g/L), cystatin C (Cys C, mg/L), systolic blood pressure (SBP, mmHg), diastolic blood pressure (DBP, mmHg), mean artery pressure (MAP, mmHg), hemoglobin (g/L), white blood cell (WBC, ×10^9^/L), uric acid (μmol/L), neutrophil gelatinaseassociated lipocalin (NGAL, ng/mL), fibrinogen (FN, g/L), D-dimer (μg/L), erythrocyte sedimentation rate (ESR, mm/h), alanine aminotransferase (ALT, U/L), aspartate aminotransferase (AST, U/L), low-density lipoprotein (LDL, mmol/L), high-density lipoprotein (HDL, mmol/L) and triglyceride (TG, mmol/L).

### Statistical analysis

Continuous variables were summarized as means ± standard deviations for normally distributed data or as medians and interquartile ranges for non-normally distributed data. Categorical variables were presented as frequencies and percentages. Comparisons between groups for quantitative variables were made using the Student t-test for normally distributed data or the Mann–Whitney U test for non-normally distributed data. Comparisons between groups for qualitative variables were conducted using chi-square or Fisher s exact tests, as appropriate. Sensitivity analysis was performed within the study cohort to compare AKI diagnosis data analysis using the KDIGO guideline alone versus the KDIGO guidelines in conjunction with expanded criteria.

Subsequently, data from the study cohort were utilized to construct a predictive model, while data form the validation set were employed to assess the model’s performance. Univariable logistic regression analysis was conducted for each variable within the study cohort. *p*-values for the variables were calculated based on the univariable logistic regression model. Variables with *p*-values <0.05 in the univariable logistic regression were included in the multivariable logistic regression model. Then, factors with *p*-values <0.05 in the multivariable model were incorporated into the prediction model to create the nomogram. In the nomogram, the sum of the assigned points for these factors, plotted on the “total points” axis, corresponded to the predicted probability ARDS occurrence-free survival in AKI patients. The discriminatory ability of the model was evaluated using receiver operating characteristic curve (ROC) analysis, and its calibration was assessed using a calibration plot for internal validation. Decision curve analysis (DCA) was applied to evaluate the clinical utility of the model. All statistical analyses were performed using SPSS (27.0 IBM, Armonk, NY, United States) and R (version 4.2.1) software.

Variables with a missing value rate of ≥20% were excluded from the analysis. The remaining variables with a missing value rate of <20% (the ratio of missing value for included variables ranged from 0 to 18.5% in total cohort; see Supplementary Table S2) were handled using multiple imputation via the mice package for R (17, 18).

## Results

### Clinical characteristics and univariate analysis results

**Figure 1:**
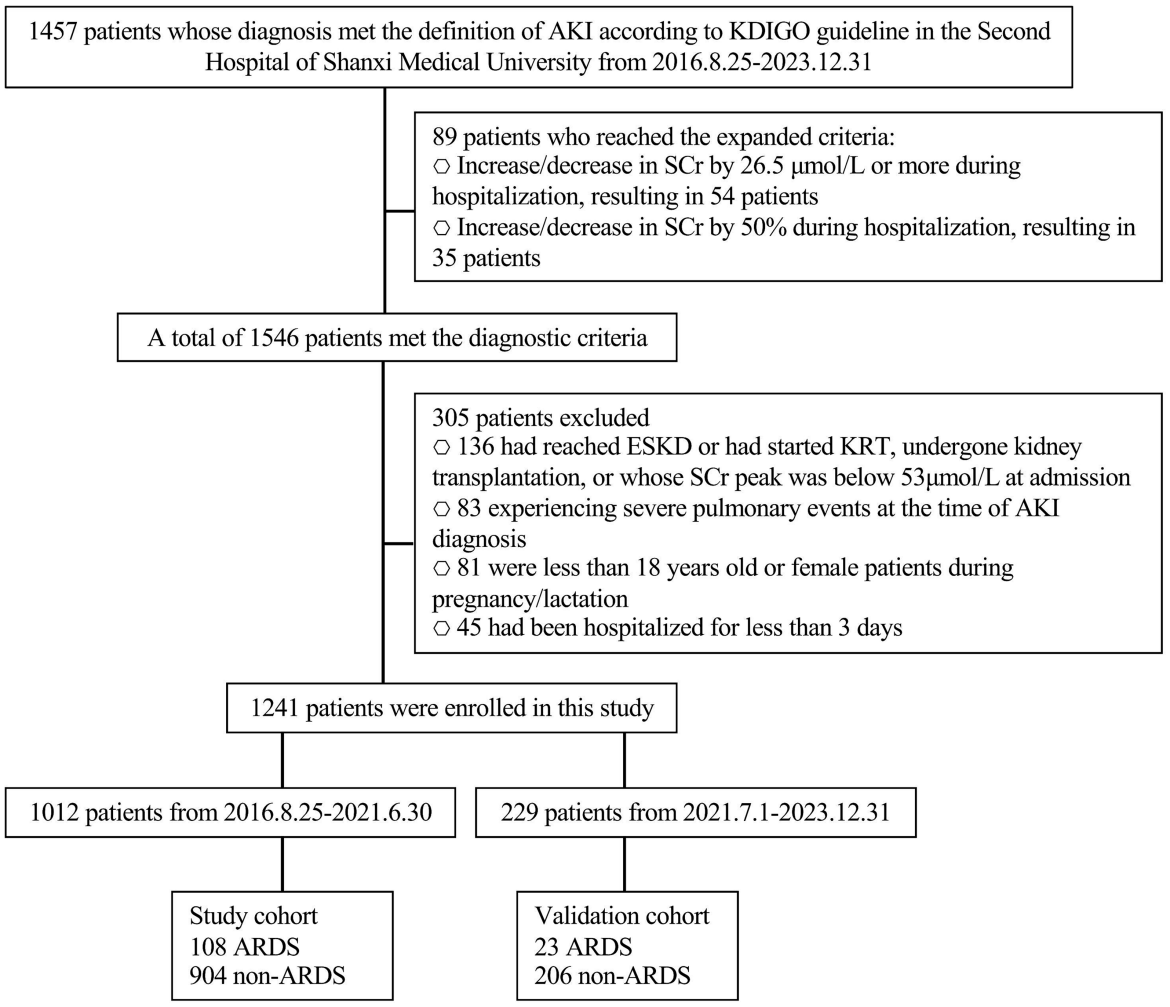
Flow chart. AKI, acute kidney injury; SCr, serum creatine; ESKD, end-stage kidney disease; KRT, kidney replacement therapy; ARDS, acute respiratory distress syndrome.

In this study, following the exclusion of ineligible cases, a total of 1,241 AKI patients who met inclusion criteria were enrolled. These patients were divided into two groups: 1012 patients comprised the study cohort enrolled between August 25, 2016, and June 30, 2021, and 229 patients comprised the validation cohort, enrolled between July 1, 2021, and December 31, 2023 (Figure 1). Among these AKI patients, 89 were diagnosed using expanded criteria, with 53 in the study cohort and 36 in the validation cohort. Of the 1,241 AKI patients, 131 (approximately 10.556%) experienced ARDS, which translates to an incidence rate of approximately 10,556 cases per 100,000 AKI patients.

Among the 1,012 patients in the study sample, 108 (10.672%) developed ARDS and were classified into the ARDS group, while 904 (89.328%) did not develop ARDS and were categorized into the non-ARDS group. The distribution and proportion of ARDS severity are presented in Table 1. The clinical characteristics of the patients are detailed in Table 2. No significant statistical differences were observed in the baseline clinicopathological data between the study and validation cohorts.

**Table 1:** ARDS severity.

ARDS severity	Cases (total = 131)	Proportion (%)
1	92	70.2
2	25	19.1
3	14	10.7

ARDS, acute respiratory distress syndrome.

**Table 2:** Characteristics of patients in the study and validation cohorts.

Characteristic	Study cohort			Comparison with validation cohort		
	ARDS group (*n* = 108)	non-ARDS (*n* = 904)	*p* value	Study cohort (*n* = 1,012)	Validation cohort (*n* = 229)	*p* value
Sex			0.584			0.652
Male	84(8.3)	341(33.7)		425(41.9)	179(43.6)	
Female	108(10.7)	479(47.3)		587(58.1)	232(56.4)	
Age (years)	56 ± 18	51 ± 17	**<0.001**	52 ± 17	52 ± 18	0.266
Smoking history			**<0.001**			0.404
No	109(10.8)	294(29.1)		403(39.8)	174(42.3)	
Yes	83(8.2)	526(52)		609(60.2)	237(57.7)	
Alcohol consumption			**0.038**			0.630
No	157(15.5)	612(60.5)		769(75.9)	318(77.4)	
Yes	35(3.5)	208(20.6)		243(24.1)	93(22.6)	
Diabetes mellitus history			**0.006**			0.875
No	173(17.1)	670(66.2)		843(83.3)	344(83.7)	
Yes	19(1.9)	150(14.8)		169(16.7)	67(16.3)	
BMI (kg/m^2^)	24.3 ± 4.3	24.8 ± 8.2	0.428	24.7 ± 7.7	24.2 ± 4.8	0.266
ALB (g/L)	28.2 ± 17.7	27.7 ± 18.2	0.753	27.8 ± 18.1	27.6 ± 15.9	0.828
Cys C (mg/L)	5.3 ± 20.5	3.6 ± 20.4	0.320	3.9 ± 20.4	4.6 ± 26.5	0.589
SBP (mmHg)	139 ± 21	124 ± 22	**<0.001**	127 ± 23	126 ± 22	0.343
DBP (mmHg)	96 ± 11	77 ± 10	**<0.001**	80 ± 13	79 ± 12	0.303
MAP (mmHg)	111 ± 10	92 ± 9	**<0.001**	96 ± 12	95 ± 12	0.193
Hemoglobin (g/L)	65 ± 58	99 ± 81	**<0.001**	72 ± 65	68 ± 56	0.441
WBC (×10^9^)	10.5 ± 3.3	9.3 ± 5.3	0.715	10.2 ± 6.1	11.5 ± 5.4	0.552
Uric acid (μmol/L)	446 ± 209	247 ± 187	**<0.001**	276 ± 155	272 ± 156	0.340
NGAL (ng/mL)	7.8 ± 1.9	10.3 ± 2.9	0.226	9.8 ± 2.6	10.3 ± 2.4	0.703
FN (g/L)	5.1 ± 1.8	3.5 ± 1.6	**<0.001**	4.9 ± 1.6	4.8 ± 1.7	0.545
D-dimer (μg/L)	2043 ± 210	688 ± 237	**<0.001**	986 ± 297	1,052 ± 269	0.543
ESR (mm/h)	155 ± 67	236 ± 64	0.100	241 ± 61	256 ± 59	0.360
LDL (mmol/L)	4.6 ± 1.7	2.1 ± 1.9	**<0.001**	4.3 ± 1.5	4.1 ± 1.7	0.646
HDL (mmol/L)	4.9 ± 1.3	1.2 ± 0.6	**<0.001**	2.8 ± 1.3	2.9 ± 1.3	0.507
TG (mmol/L)	3.8 ± 2.7	2.3 ± 1.4	**<0.001**	2.5 ± 2.6	2.7 ± 1.4	0.389
ALT (U/L)	38.4 ± 17.3	37.2 ± 18.1	0.827	36.9 ± 18.5	38.5 ± 17.8	0.797
AST (U/L)	39.1 ± 15.7	31 ± 12.2	0.340	32.7 ± 15.3	33.9 ± 12.7	0.840
AKI stage			**<0.001**			0.957
Stage 1	96(9.5)	779(77.1)		875(86.5)	353(85.9)	
Stage 2	41(4.1)	35(3.5)		76(7.5)	31(7.5)	
Stage 3	55(5.4)	5(0.5)		60(5.9)	27(6.6)	
AKI cause			0.238			0.865
Pre-renal AKI	45(4.4)	153(15.1)		198(19.6)	87(21.2)	
Intrinsic AKI	45(4.4)	246(24.3)		291(28.8)	114(27.7)	
Postrenal AKI	33(3.3)	139(13.7)		172(16.9)	73(17.8)	
Unclassified	69(6.8)	282(27.9)		351(34.7)	137(33.3)	
Injury factors			**<0.001**			0.844
DCC	8(0.8)	42(4.2)		50(4.9)	25(6.1)	
RAS or RAO	16(1.6)	30(3.0)		46(4.5)	20(4.9)	
Hemorrhage	21(2.1)	73(7.2)		94(9.3)	38(9.2)	
AGE	12(1.2)	8(0.8)		20(1.9)	8(1.9)	
Nephrotoxic drugs/toxins	33(3.3)	234(23.1)		267(26.4)	107(26)	
CIN	6(0.6)	12(1.2)		18(1.8)	7(1.7)	
Sepsis	45(4.4)	208(20.6)		253(25)	95(23.1)	
Surgery	12(1.2)	46(4.5)		58(5.7)	24(5.8)	
Rhabdomyolysis	6(0.6)	28(2.8)		34(3.4)	14(3.4)	
Urinary tract calculi or stenosis	15(1.5)	82(8.1)		97(9.6)	38(9.2)	
Prostatauxe	10(1.0)	32(3.2)		42(4.2)	20(4.9)	
Urologic/pelvic cancer	4(0.4)	16(1.6)		20(1.9)	9(2.2)	
Neurogenic bladder	4(0.4)	9(0.9)		13(1.3)	6(1.4)	

BMI, body mass index; ALB, albumin; Cys C, cystatin C; SBP, systolic blood pressure; DBP, diastolic blood pressure; MAP, mean artery pressure; WBC, white blood cell; NGAL, neutrophil gelatinase-associated lipocalin; FN, fibronectin; ESR, erythrocyte sedimentation rate; LDL, low-density lipoprotein; HDL, high-density lipoprotein; TG, triglyceride; ALT, alanine aminotransferase; AST, aspartate aminotransferase; DCC, decompensated cirrhosis; RAS, renal artery stenosis; RAO, renal artery occlusion; AGE, acute gastroenteritis; CIN, contrast-induced nephropathy. *p* < 0.05 was statistically significant. All *P*-values < 0.05 are bolded to highlight statistical significance for better readability.

Univariate logistic regression analysis of the study cohort revealed that age, history, alcohol consumption, diabetes mellitus history, SBP, DBP, MAP, hemoglobin, uric acid, FN, D-dimer, LDL, HDL, TG, AKI stage, and injury factors were significantly different between AKI patients with and without ARDS (*p* < 0.05). Specifically, patients with ARDS exhibited higher levels of age, SBP, DBP, MAP, uric acid, FN, D-dimer, LDL, HDL, and TG compared to those without ARDS (mean age: 56 ± 18 vs. 51 ± 17; mean SBP level: 139 ± 21 vs. 124 ± 22; mean DBP level: 96 ± 11 vs. 77 ± 10; mean MAP level: 111 ± 10 vs. 92 ± 9; mean uric acid level: 446 ± 209 vs. 247 ± 187; mean FN level: 5.1 ± 1.8 vs. 3.5 ± 1.6; mean D-dimer level: 2043 ± 210 vs. 688 ± 237; mean LDL level: 4.6 ± 1.7 vs. 2.1 ± 1.9; mean HDL level: 4.9 ± 1.3 vs. 1.2 ± 0.6; mean TG level: vs. 3.8 ± 2.7 vs. 2.3 ± 1.4), while hemoglobin levels were lower in patients with ARDS (mean hemoglobin level: 65 ± 58 vs. 99 ± 81), all with *p* values <0.05. Categorical variables such as AKI stage and injury factors also demonstrated significant difference between patients with and without ARDS (AKI stage: *p* < 0.001; injury factor: *p* < 0.001).

### Multivariate analysis

The significant factors identified in the univariate logistic analysis were incorporated into a multivariate logistic regression model to determine whether each factor was an independent risk factor for the development of ARDS. Among these factors, MAP, SBP and DBP were all significantly different. We chose to include MAP rather than SBP and DBP in the model, as MAP provides a more comprehensive description blood pressure status. In the multivariate analysis, the following factors were found to be independently associated with ARDS, with *p* values <0.05: older age (OR = 1.020; 95%CI = 1.005–1.036), smoking history (OR = 1.416; 95%CI = 1.213–1.811), history of diabetes mellitus (OR = 1.449; 95%CI = 1.202–1.797), higher MAP (OR = 1.165; 95%CI = 1.132–1.199), elevated uric acid level (OR = 1.002; 95%CI = 1.001–1.004), and higher AKI stage [(stage 1: reference), (stage 2: OR = 11.863; 95%CI = 4.850–29.014), (stage 3: OR = 41.398; 95%CI = 30.840–52.731)]. These results are detailed in Table 3.

**Table 3:** Multivariate logistic analysis of the study cohort.

Characteristic	OR (95%CI)	*p* value
Age (years)	1.020(1.005–1.036)	**0.008**
Smoking history (yes)	1.416(1.213–1.811)	**0.010**
Alcohol consumption (yes)	1.034(0.474–2.254)	0.934
Diabetes mellitus history (yes)	1.449(1.202–1.797)	**0.049**
MAP (mmHg)	1.165(1.132–1.199)	**<0.001**
Hemoglobin (g/L)	1.001(0.996–1.006)	0.662
Uric acid (μmol/L)	1.002(1.001–1.004)	**0.002**
FN (g/L)	1.092(0.933–1.280)	0.274
D-dimer (μg/L)	1.210(1.017–1.612)	0.705
LDL (mmol/L)	1.003(0.964–1.043)	0.876
HDL (mmol/L)	1.683(1.440–2.061)	0.089
TG (mmol/L)	1.076(1.001–1.219)	0.250
AKI stage		
Stage 1	Reference	
Stage 2	11.863(4.850–29.014)	**<0.001**
Stage 3	41.398(30.840–52.731)	**<0.001**
Injury factors		0.618
DCC	Reference	
RAS or RAO	2.022(0.379–10.792)	0.516
Hemorrhage	1.664(0.358–7.731)	0.169
AGE	3.837(0.565–26.054)	0.822
Nephrotoxic drugs/toxins	1.178(0.283–4.906)	0.072
CIN	7.203(0.836–62.073)	0.388
Sepsis	1.869(0.452–7.726)	0.358
Surgery	2.188(0.412–11.606)	0.552
Rhabdomyolysis	1.779(0.267–11.853)	0.798
Urinary tract calculi or stenosis	1.232(0.249–6.106)	0.443
Prostatauxe	2.034(0.332–12.457)	0.537
Urologic/pelvic cancer	2.448(0.142–42.106)	0.092

MAP, mean artery pressure. FN, fibronectin; LDL, low-density lipoprotein; HDL, high-density lipoprotein; TG, triglyceride; AKI, acute kidney injury; DCC, decompensated cirrhosis; RAS, renal artery stenosis; RAO, renal artery occlusion; AGE, acute gastroenteritis; CIN, contrast-induced nephropathy. *p* < 0.05 was statistically significant. All *P*-values < 0.05 are bolded to highlight statistical significance for better readability.

To further substantiate the robustness of our findings, particularly in light of the inclusion of expanded criteria, a sensitivity analysis was conducted. The results of this analysis revealed that the conclusions remained largely consistent, with no significant variations observed (Table 4). This analysis thus provides additional assurance regarding the stability and reliability of our study outcomes.

**Table 4:** Comparing data analysis in study cohort: AKI diagnosis using KDIGO Guidelines vs. KDIGO Guidelines with expanded criteria.

Characteristic	With expanded criteria (*n* = 1,012)		Without expanded criteria (*n* = 959)	
	Univariate analysis *p*-value [OR(95% CI)]	Multivariate analysis *p*-value [OR(95% CI)]	Univariate analysis *p*-value [OR(95% CI)]	Multivariate analysis *p*-value [OR(95% CI)]
Sex (Male/female)	0.584 [0.915 (0.667–1.257)]		0.493 [1.195 (0.718–1.990)]	
Age (years)	<0.001 [1.020 (1.011–1.030)]	0.008 [1.020(1.005–1.036)]	0.076 [1.013 (0.999–1.028)]	0.024 [1.020 (0.994–1.047)]
Smoking history (No/Yes)	<0.001 [1.426 (1.309–0.586)]	0.010 [1.416(1.213–1.811)]	<0.001 [1.362 (1.216–1.607)]	0.006 [1.300 (1.087–1.714)]
Alcohol consumption (No/Yes)	0.038 [0.656 (0.440–0.977)]	0.934 [1.034(0.474–2.254)]	0.228 [0.671 (0.351–1.283)]	0.716 [0.771 (0.190–3.125)]
Diabetes mellitus history (No/Yes)	0.006 [0.049 (0.296–0.813)]	0.049 [1.449(1.202–1.797)]	0.076 [0.474 (0.207–1.082)]	0.038 [1.554 (1.148–2.078)]
BMI (kg/m2)	0.428 [0.988 (0.961–1.017)]		0.552 [0.983 (0.929–1.040)]	
ALB (g/L)	0.753 [1.001 (0.993–1.010)]		0.740 [1.003 (0.988–1.018)]	
Cys C (mg/L)	0.320 [1.003 (0.997–1.010)]		0.404 [1.003 (0.996–1.011)]	
SBP (mmHg)	<0.001 [1.030 (1.023–1.037)]		<0.001 [1.031 (1.020–1.043)]	
DBP (mmHg)	<0.001 [1.214 (1.179–1.250)]		<0.001 [1.191 (1.143–1.241)]	
MAP (mmHg)	<0.001 [1.175 (1.149–1.201)]	<0.001 [1.165(1.132–1.199)]	<0.001 [1.172 (1.131–1.214)]	<0.001 [1.006 (1.002–1.009)]
Hemoglobin (g/L)	<0.001 [1.010 (1.007–1.013)]	0.662 [1.001(0.996–1.006)]	<0.001 [1.009 (1.004–1.014)]	0.088 [0.987 (0.973–1.002)]
WBC (×109)	0.715 [0.997 (0.978–1.015)]		0.793 [0.995 (0.957–1.034)]	
Uric acid (μmol/L)	<0.001 [1.008 (1.003–1.014)]	0.002 [1.012(1.001–1.024)]	<0.001 [1.009 (1.003–1.015)]	<0.001 [1.006 (1.002–1.009)]
NGAL (ng/mL)	0.226 [0.996 (0.988–1.003)]		0.811 [0.999 (0.989–1.009)]	
FN (g/L)	<0.001 [0.834 (0.756–0.920)]	0.274 [1.092(0.933–1.280)]	0.036 [0.848 (0.727–0.989)]	0.170 [1.228 (0.915–1.648)]
D-dimer (μg/L)	<0.001 [1.013 (1.001–1.026)]	0.705 [1.210(1.017–1.612)]	0.007 [1.017 (1.001–1.094)]	0.608 [1.035 (1.012–1.067)]
ESR (mm/h)	0.100 [1.001 (0.999–1.004)]		0.579 [1.035 (1.013–1.057)]	
LDL (mmol/L)	<0.001 [1.762 (1.688–1.844)]	0.876 [1.003(0.964–1.043)]	<0.001 [1.685 (1.571–1.822)]	0.988 [1.001 (0.911–1.113)]
HDL (mmol/L)	<0.001 [1.400 (1.313–1.510)]	0.089 [1.683(1.440–2.061)]	<0.001 [1.384 (1.261–1.565)]	0.120 [0.554 (0.264–1.165)]
TG (mmol/L)	<0.001 [1.720 (1.633–1.818)]	0.250 [1.076(1.001–1.219)]	0.001 [1.712 (1.581–1.874)]	0.802 [1.056 (0.689–1.619)]
ALT (U/L)	0.827 [1.056 (1.001–1.154)]		0.720 [0.999 (0.996–1.003)]	
AST (U/L)	0.340 [2.127 (2.089–2.513)]		0.635 [1.076 (1.001–1.112)]	
AKI stage				
Stage 1	Reference	Reference	Reference	Reference
Stage 2	<0.001 [9.506 (5.775–15.647)]	<0.001 [11.863(4.850–29.014)]	<0.001 [6.827 (3.119–14.942)]	0.004 [9.606 (2.095–14.501)]
Stage 3	<0.001 [59.260 (34.877–61.737)]	<0.001 [41.398(30.840–52.731)]	<0.001 [33.553 (28.221–36.669)]	<0.001 [21.645 (10.310–38.579)]
AKI cause				
Pre-renal AKI	Reference		Reference	
Intrinsic AKI	0.043 [0.622 (0.393–0.985)]		0.019 [0.414 (0.197–0.866)]	
Postrenal AKI	0.405 [0.807 (0.487–1.377)]		0.769 [0.897 (0.434–1.855)]	
Unclassified	0.395 [0.832 (0.544–1.271)]		0.092 [0.565 (0.291–1.099)]	
Injury factors				
DCC	Reference	Reference	Reference	Reference
RAS or RAO	0.368 [1.510 (0.615–3.709)]	0.516 [2.022(0.379–10.792)]	0.618 [1.400 (0.868–14.110)]	0.839 [1.271 (0.126–12.807)]
Hemorrhage	0.001 [7.875 (2.441–25.406)]	0.169 [1.664(0.358–7.731)]	0.005 [15.750 (2.298–19.717)]	0.598 [2.299 (0.104–5.953)]
AGE	0.483 [0.740 (0.320–1.714)]	0.822 [3.837(0.565–26.054)]	0.606 [1.726 (1.215–2.451)]	0.453 [0.448 (0.055–3.649)]
Nephrotoxic drugs/toxins	0.126 [2.625 (0.761–9.051)]	0.072 [1.178(0.283–4.906)]	0.912 [2.875 (1.082–9.376)]	0.854 [1.504 (1.203–4.239)]
CIN	0.761 [1.136 (0.499–2.594)]	0.388 [7.203(0.836–62.073)]	0.768 [1.832 (1.246–2.816)]	0.838 [1.802 (1.096–6.687)]
Sepsis	0.533 [1.370 (0.510–3.677)]	0.358 [1.869(0.452–7.726)]	0.727 [1.750 (1.149–3.769)]	0.588 [0.462 (0.028–7.524)]
Surgery	0.842 [1.125 (0.352–3.594)]	0.552 [2.188(0.412–11.606)]	0.887 [0.875 (0.139–5.507)]	0.845 [0.740 (0.036–10.080)]
Rhabdomyolysis	0.932 [0.960 (0.377–2.447)]	0.798 [1.779(0.267–11.853)]	0.618 [1.400 (0.373–5.259)]	0.933 [0.902 (0.081–10.041)]
Urinary tract calculi or stenosis	0.350 [1.641 (0.581–4.629)]	0.443 [1.232(0.249–6.106)]	0.149 [2.827 (0.690–11.577)]	0.289 [3.725 (1.327–4.396)]
Prostatauxe	0.689 [1.313 (0.347–4.969)]	0.537 [2.034(0.332–12.457)]	0.724 [0.656 (0.063–6.797)]	0.724 [2.946 (1.107–8.851)]
Urologic/pelvic cancer	0.235 [2.333 (0.576–9.458)]	0.092 [2.448(0.142–42.106)]	0.958 [1.050 (0.095–11.558)]	0.663 [3.190 (1.017–6.261)]

AKI, acute kidney injury; BMI, body mass index; ALB, albumin; Cys C, cystatin C; SBP, systolic blood pressure; DBP, diastolic blood pressure; MAP, mean artery pressure; WBC, white blood cell; NGAL, neutrophil gelatinase-associated lipocalin; FN, fibronectin; ESR, erythrocyte sedimentation rate; LDL, low-density lipoprotein; HDL, high-density lipoprotein; TG, triglyceride; ALT, alanine aminotransferase; AST, aspartate aminotransferase; DCC, decompensated cirrhosis; RAS, renal artery stenosis; RAO, renal artery occlusion; AGE, acute gastroenteritis; CIN, contrast-induced nephropathy. *p* < 0.05 was statistically significant.

### Performance of the nomogram

**Figure 2:**
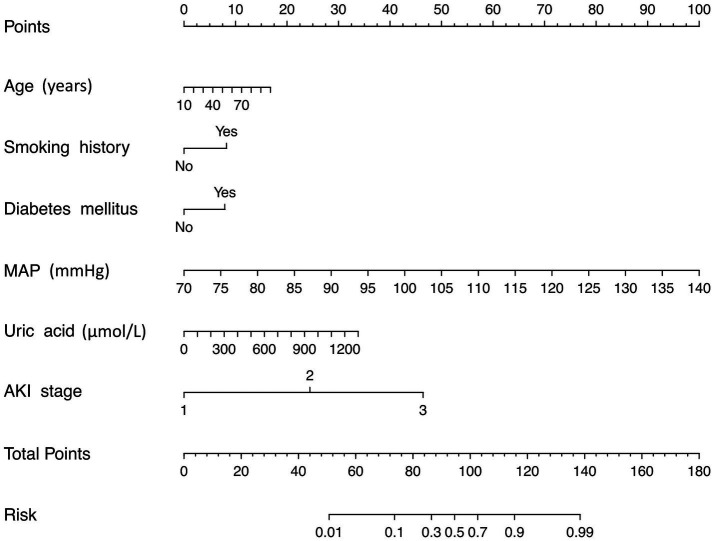
Risk nomogram conducted by logistic regression. MAP, mean artery pressure; AKI, acute kidney injury.

**Figure 3:**
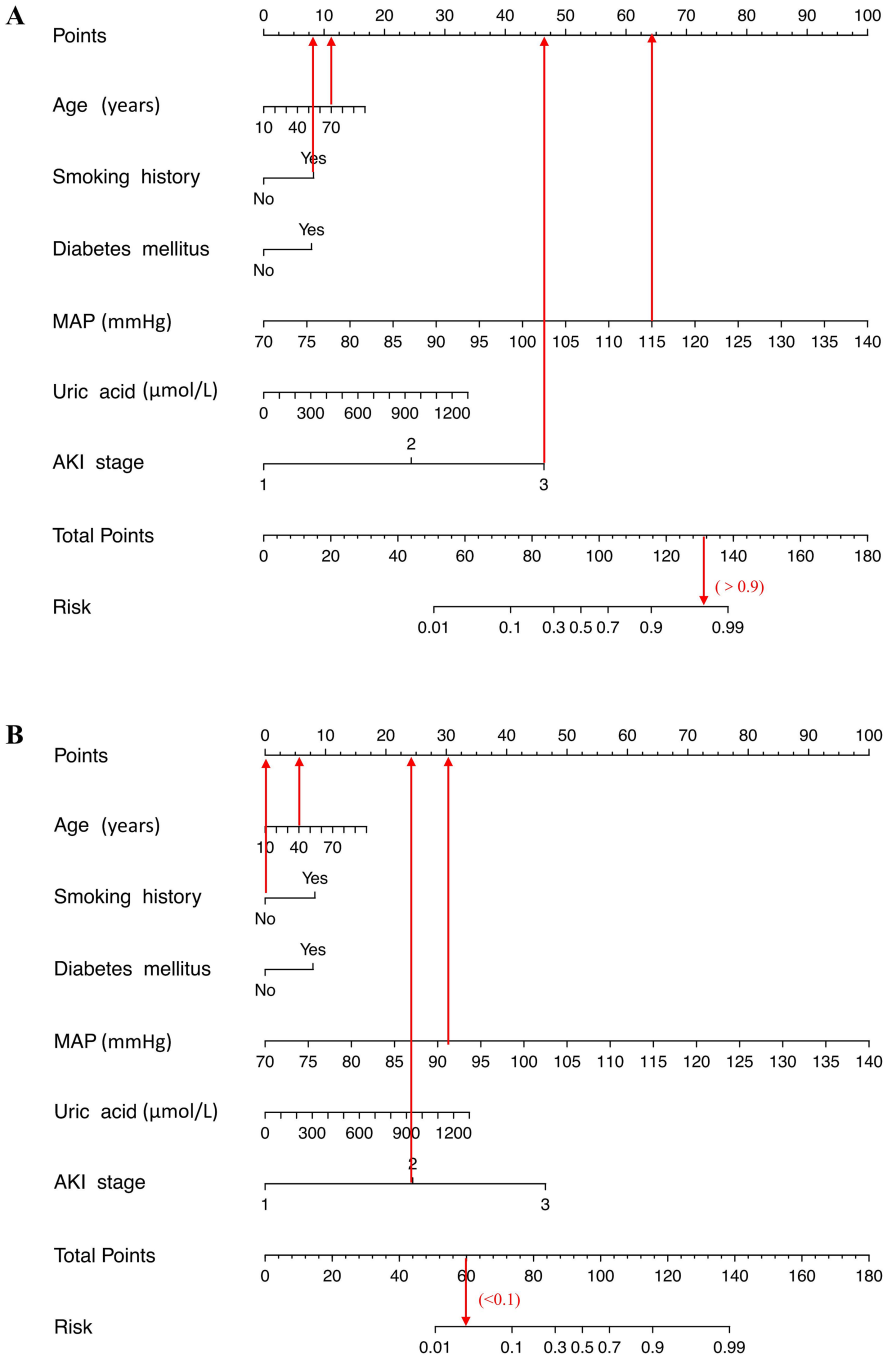
Examples of the nomogram in clinical practice. Figures illustrate the process of calculating the risk scores of acute respiratory distress syndrome (ARDS) and non-ARDS using the nomogram. **(A)** A 70-year-old patient with a history of smoking, a mean artery pressure (MAP) of 115 mmHg, and an AKI stage of 3 have a risk index exceeding 0.9 (90%). **(B)** A 40-year-old patient with no smoking history, MAP level of 91 mmHg, and stage 2 of acute kidney injury (AKI) at admission, which corresponds to an ARDS risk of less than 0.1 (<10%).

A risk estimation nomogram was constructed utilizing the 6 independent risk factors identified, based on the probability of remaining free from ARDS occurrence (Figure 2). In the nomogram, each selected predictor is assigned a score according to the values derived from the prediction model. To determine the total score, a vertical line is drawn from the value of each predictor perpendicular to the points axis. The score corresponding to each predictor is indicated by the intersection on the points axis. For instance, a 70-year-old patient with a history of smoking, a MAP of 115 mmHg, and an AKI stage of 3 have a risk index exceeding 0.9 (Figure 3A). Another illustrative example is provided in Figure 3B.

We recommend that AKI patients presenting with advanced age, a history of smoking, diabetes mellitus, elevated MAP, high uric acid levels, and a high AKI stage should undergo regular medical monitoring and receive appropriate treatment to mitigate the risk of developing ARDS.

### Validation and effect evaluation of the nomogram

**Figure 4:**
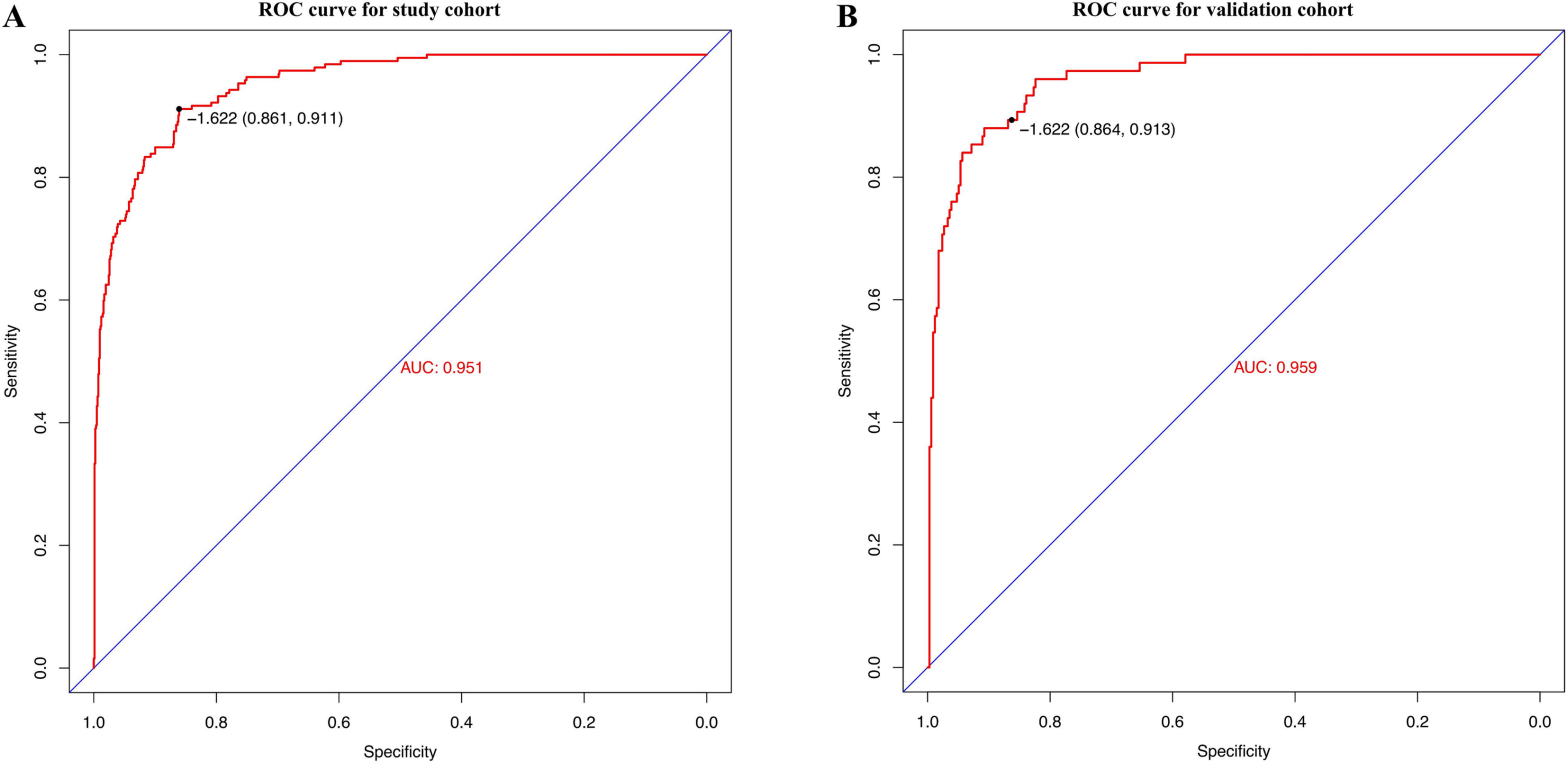
Receiver operating characteristic (ROC) curves of the nomogram in the study cohort **(A)** and the validation cohort **(B)**.

**Figure 5:**
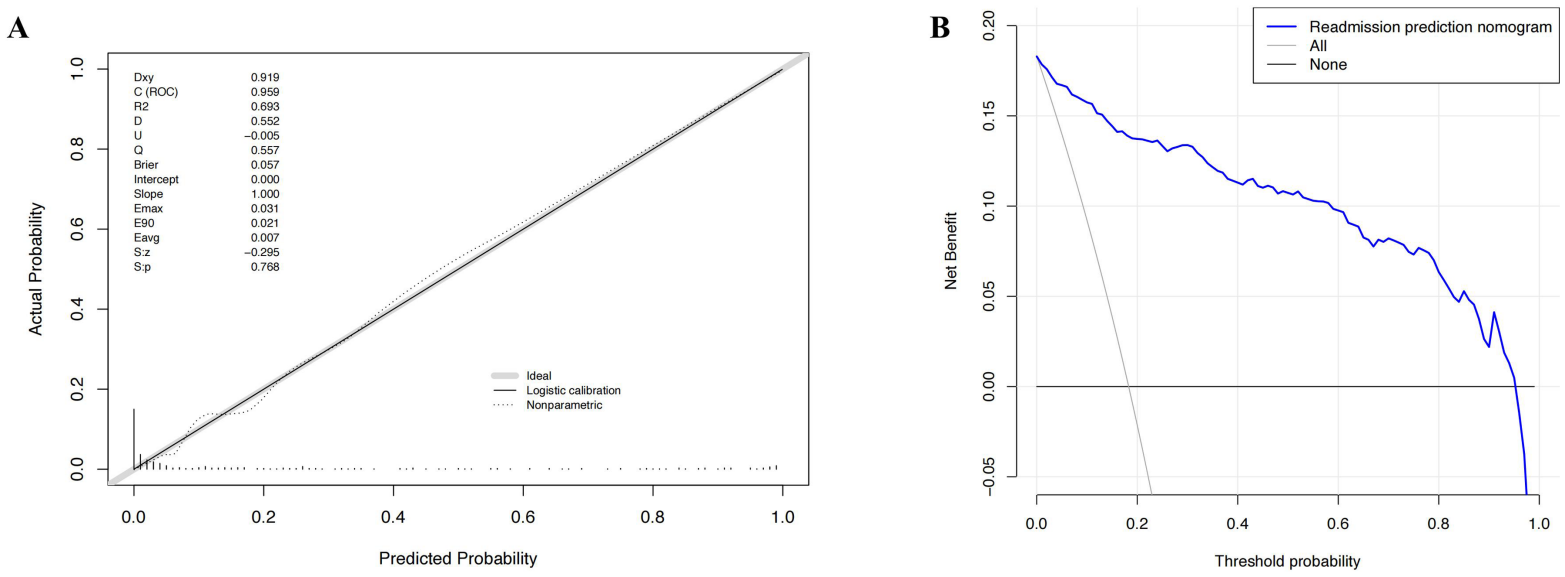
The calibration curve and results of the decision curve analysis (DCA) of the nomogram in the validation cohort. **(A)** Calibration curves represent the difference between the actual prediction and the ideal perfect prediction (45° line). **(B)** The DCA curve of the nomogram for predicting acute respiratory distress syndrome (ARDS). It revealed that the nomogram could obtain a greater net benefit than either the “treat all” or the “treat none” strategy.

The developed model underwent internal validation utilizing the validation cohort. The discriminatory performance of the nomogram was assessed by the ROC curve (Figure 4). The nomogram demonstrated an AUC of 0.951 (Figure 4A). In the validation cohort, the nomogram maintained robust discriminatory ability, with an AUC of 0.959 (Figure 4B). Utilizing the cutoff value (−1.622) derived from the ROC analysis of the study cohort, the specificity and sensitivity of the prediction model in the validation cohort were 86.4 and 91.3%, respectively, closely aligning with the study cohort’s results of 86.1 and 91.1% (Figure 4; Table 5). The calibration curve revealed satisfactory agreement between the nomogram’s predictions the actual observation (Figure 5A). The DCA indicated that the prediction model offers substantial net benefit, facilitating valuable and clinically beneficial decision-making (Figure 5B).

**Table 5:** Prediction model accuracy in the validation cohort.

Prediction	Actual observation	
	ARDS	Non-ARDS
ARDS	21	22
Non-ARDS	7	178
	23	206
Sensitivity of model: 91.3% (21/23)		
Specificity of model: 86.4% (178/206)		

ARDS, acute respiratory distress syndrome.

## Discussion

In this retrospective study, we examined 1,241 AKI patients hospitalized in our facility, of whom 131 subsequently developed ARDS. Our results identified older age, smoking history, history of diabetes mellitus, MAP, higher uric acid level, and higher AKI stage as independent risk factors ARDS following AKI. The nomogram developed in this study offers a practical tool for assessing the risk of ARDS in patients with AKI by incorporating these key clinical variables. In clinical practice, clinicians can use this nomogram by inputting these factors to calculate a risk score, which can assist in identifying high-risk patients. Those with higher risk scores may require more intensive monitoring, while lower-risk patients can be monitored less frequently.

Advanced age is correlated with structural alterations and functional deterioration of the lungs, and it is a well-established risk factor for ARDS (19). The incidence of ARDS increases by 3% for every decade of life (20). Observational cohort studies have indicated that ARDS patients aged 65 years or older have a significantly lower survival rate compared to those under 65 years (21). However, the influence of age on the development of ARDS following AKI remains unclear. Our study demonstrated that age is an independent predictor of respiratory failure following AKI. Cigarette smoke exposure has been recognized as a risk factor for ARDS across diverse populations. Both active and passive smoking are linked to an increased vulnerability to lung injury in patients with sepsis and blunt trauma (22, 23). Our study corroborated these findings, concluding that smoking is associated with ARDS in a cohort of AKI patients. Furthermore, based on prior research, not only active smokers but also individuals chronically exposed to secondhand smoke should be considered at heightened risk clinical practice. A prior meta-analysis revealed that diabetes mellitus is significantly associated with an increased incidence of ARDS in hospitalized patients (24). Patients with renal insufficiency and concomitant diabetes mellitus exhibit compromised pulmonary function. They face an elevated risk of ESRD and a higher mortality risk (25). Our study further corroborated that diabetic patients are significantly more likely to develop ARDS following AKI compared to non-diabetic patients. Our research also indicated that AKI patients with higher MAP are at a greater risk of developing ARDS. Hypertension, in conjunction with a systemic inflammatory response characterized by elevated high-sensitivity C-reactive protein, various cytokines and complement activation (26), may facilitate the kidney-lung crosstalk process, thereby increasing susceptibility to lung injury. Uric acid has been proposed as a prognostic predictor of ARDS and is the most abundant antioxidant in human serum. A previous large-scale cohort study demonstrated that serum uric acid levels are inversely associated with the risk of respiratory disease (27). Moreover, survival rates were significantly lower in the hyperuricemia group (28, 29). Our findings suggest that uric acid is also an underrecognized risk factor for the development of ARDS following AKI. Given its simplicity, low cost, and ready availability, uric acid should be emphasized as a biomarker in the management of patients with AKI. Our study demonstrated that patients with AKI stage 3 were at a heightened risk of lung injury compared to other patients. The clinical presentation of patients with AKI stage 3 is marked by a rapid escalation in SCr over a brief period or a reduction in urine output, which can lead to increased renal-pulmonary cross-infection and a heightened risk of respiratory failure (8). Consistent with our findings, among AKI patients with concomitant ARDS, kidney outcomes were more adverse in stage 2 and 3 patients compared to those in stage 1 (30). This suggests that the severity of AKI is a significant predictor of respiratory failure, and patients with severe AKI should be closely monitored for the development of ARDS.

In our study, we endeavored to calculate all the relevant risk factors and the elucidate the relationship between these risk factors and development of ARDS following AKI. However, like other retrospective studies, our study has limitations. Firstly, selection bias is inherent and unavoidable. We utilized e Berlin definition as the ARDS inclusion. However, this definition may not be applicable in scenarios where arterial blood gas data are difficult to obtain, potentially leading to the under-identification of some patients (31). Our sensitivity analysis, which showed that the conclusions remained largely consistent, further supports the stability of our study’s inclusion criteria. Secondly, studies spanning long durations are prone to lose some follow-up data, which can introduce bias. Our data collection commenced in August 2016, and there has been a considerable number of patients have become unreachable, resulting in missing information. Thirdly, the limited sample size may have impacted the study’s quality. We only included the patients from the Department of Nephrology of our hospital, which makes this a single-center study. Consequently, the results may not be fully generalizable to other populations or institutions. Variations in patient characteristics, clinical practices, and institutional protocols could influence the observed outcomes. To confirm the accuracy of the identified risk factors and the nomogram, a larger, multi-center cohort study is necessary in the next phase.

## Conclusion

The findings our study indicate that older age, smoking history, history of diabetes mellitus, MAP, higher serum uric acid level, and higher AKI stage are independent risk factors for development or ARDS following AKI. In clinical practice, heightened attention to these risk factors, coupled with early identification and timely intervention, may help reduce the incidence of ARDS and improve the prognosis for patients with AKI.

## Data Availability

The datasets presented in this article are not readily available because data from this study may contain potentially or sensitive patient information. Requests to access the datasets should be directed to qiaoxi7347@vip.163.com.

## References

[1] Mehta RL Cerdá J Burdmann EA Tonelli M García-García G Jha V. International Society of Nephrology's 0by25 initiative for acute kidney injury (zero preventable deaths by 2025): a human rights case for nephrology. Lancet. (2015) 385:2616–43. doi: 10.1016/S0140-6736(15)60126-X, PMID: 25777661

[2] Shiao CC Wu PC Huang TM Lai TS Yang WS Wu CH. Long-term remote organ consequences following acute kidney injury. Crit Care. (2015) 19:438. doi: 10.1186/s13054-015-1149-5, PMID: 26707802 PMC4699348

[3] Al-Jaghbeer M Dealmeida D Bilderback A Ambrosino R Kellum JA. Clinical decision support for in-hospital AKI. J Am Soc Nephrol. (2018) 29:654–60. doi: 10.1681/ASN.2017070765, PMID: 29097621 PMC5791078

[4] Dépret F Prud'homme M Legrand M. A role of remote organs effect in acute kidney injury outcome. Nephron. (2017) 137:273–6. doi: 10.1159/000476077, PMID: 28586768

[5] Husain-Syed F Rosner MH Ronco C. Distant organ dysfunction in acute kidney injury. Acta Physiol (Oxford). (2020) 228:e13357. doi: 10.1111/apha.13357 31379123

[6] Herrlich A. Interorgan crosstalk mechanisms in disease: the case of acute kidney injury-induced remote lung injury. FEBS Lett. (2022) 596:620–37. doi: 10.1002/1873-3468.14262, PMID: 34932216

[7] Tomasi A Song X Gajic O Kashani K. Kidney and lung crosstalk during critical illness: large-scale cohort study. J Nephrol. (2023) 36:1037–46. doi: 10.1007/s40620-022-01558-9, PMID: 36692665

[8] Lee SA Cozzi M Bush EL Rabb H. Distant organ dysfunction in acute kidney injury: a review. Am J Kidney Dis. (2018) 72:846–56. doi: 10.1053/j.ajkd.2018.03.028, PMID: 29866457 PMC6252108

[9] Domenech P Perez T Saldarini A Uad P Musso CG. Kidney-lung pathophysiological crosstalk: its characteristics and importance. Int Urol Nephrol. (2017) 49:1211–5. doi: 10.1007/s11255-017-1585-z, PMID: 28401379

[10] Fadel RA Murskyj I Abou Asala E Nasiri N Alsaadi A Scott A. Oliguria on the day of intubation is associated with mortality in patients with acute respiratory distress syndrome. Crit Care Explor. (2022) 4:e0717. doi: 10.1097/CCE.0000000000000717, PMID: 35747122 PMC9208888

[11] Yang L Xing G Wang L Wu Y Li S Xu G. Acute kidney injury in China: a cross-sectional survey. Lancet. (2015) 386:1465–71. doi: 10.1016/S0140-6736(15)00344-X, PMID: 26466051

[12] Kdigo K. KDIGO clinical practice guideline for acute kidney injury. Kidney Int Suppl. (2012) 1:1–138. Available at: https://kdigo.org/guidelines/acute-kidney-injury/

[13] World Health Organization ed. ICD-10: International statistical classification of diseases and related health problems: Tenth revision. 2nd ed. Geneva, Switzerland: World Health Organization (2004).

[14] von Elm E Altman DG Egger M Pocock SJ Gøtzsche PC Vandenbroucke JP. The strengthening the reporting of observational studies in epidemiology (STROBE) statement: guidelines for reporting observational studies. J Clin Epidemiol. (2008) 61:344–9. doi: 10.1016/j.jclinepi.2007.11.008, PMID: 18313558

[15] ARDS Definition Task Force Ranieri VM Rubenfeld GD. Acute respiratory distress syndrome: the Berlin Definition. JAMA. (2012) 307:2526–33. doi: 10.1001/jama.2012.5669, PMID: 22797452

[16] Lameire N Van Biesen W Vanholder R. Acute renal failure. Lancet. (2005) 365:417–30. doi: 10.1016/S0140-6736(05)17831-3 15680458

[17] Dusseux E Albano L Fafin C Hourmant M Guérin O Couchoud C. A simple clinical tool to inform the decision-making process to refer elderly incident dialysis patients for kidney transplant evaluation. Kidney Int. (2015) 88:121–9. doi: 10.1038/ki.2015.25, PMID: 25671769

[18] de Goeij MC van Diepen M Jager KJ Tripepi G Zoccali C Dekker FW. Multiple imputation: dealing with missing data. Nephrol Dial Transplant. (2013) 28:2415–20. doi: 10.1093/ndt/gft221, PMID: 23729490

[19] Ely EW Wheeler AP Thompson BT Ancukiewicz M Steinberg KP Bernard GR. Recovery rate and prognosis in older persons who develop acute lung injury and the acute respiratory distress syndrome. Ann Intern Med. (2002) 136:25–36. doi: 10.7326/0003-4819-136-1-200201010-00007, PMID: 11777361

[20] Kasotakis G Stanfield B Haines K Vatsaas C Alger A Vaslef SN. Acute respiratory distress syndrome (ARDS) after trauma: improving incidence, but increasing mortality. J Crit Care. (2021) 64:213–8. doi: 10.1016/j.jcrc.2021.05.003, PMID: 34022661

[21] Kao KC Hsieh MJ Lin SW Chuang LP Chang CH Hu HC. Survival predictors in elderly patients with acute respiratory distress syndrome: a prospective observational cohort study. Sci Rep. (2018) 8:13459. doi: 10.1038/s41598-018-31811-w, PMID: 30194437 PMC6128868

[22] Calfee CS Matthay MA Eisner MD Benowitz N Call M Pittet JF. Active and passive cigarette smoking and acute lung injury after severe blunt trauma. Am J Respir Crit Care Med. (2011) 183:1660–5. doi: 10.1164/rccm.201011-1802OC, PMID: 21471091 PMC3136993

[23] Moazed F Hendrickson C Jauregui A Gotts J Conroy A Delucchi K. Cigarette smoke exposure and acute respiratory distress syndrome in Sepsis: epidemiology, clinical features, and biologic markers. Am J Respir Crit Care Med. (2022) 205:927–35. doi: 10.1164/rccm.202105-1098OC, PMID: 35050845 PMC9838633

[24] Bradley SA Banach M Alvarado N Smokovski I Bhaskar SMM. Prevalence and impact of diabetes in hospitalized COVID-19 patients: a systematic review and meta-analysis. J Diabetes. (2022) 14:144–57. doi: 10.1111/1753-0407.13243, PMID: 34939735 PMC9060142

[25] Patel I Gong HJ Xu H Chai YH Qiao YS Zhang JY. Association between measures of kidney function and preserved ratio impaired spirometry in diabetes: NHANES 2007-2012. BMJ Open. (2024) 14:e075955. doi: 10.1136/bmjopen-2023-075955, PMID: 39486815 PMC11529460

[26] Xiao L Harrison DG. Inflammation in hypertension. Can J Cardiol. (2020) 36:635–47. doi: 10.1016/j.cjca.2020.01.013, PMID: 32389337 PMC7733126

[27] Horsfall LJ Nazareth I Petersen I. Serum uric acid and the risk of respiratory disease: a population-based cohort study. Thorax. (2014) 69:1021–6. doi: 10.1136/thoraxjnl-2014-205271, PMID: 24904021 PMC4215274

[28] Lee HW Choi SM Lee J Park YS Lee CH Yim JJ. Serum uric acid level as a prognostic marker in patients with acute respiratory distress syndrome. J Intensive Care Med. (2019) 34:404–10. doi: 10.1177/0885066617698911, PMID: 28351229

[29] Pehlivanlar-Kucuk M Kucuk AO Ozturk CE Er MC Ulger F. The association between serum uric acid level and prognosis in critically ill patients, uric acid as a prognosis predictor. Clin Lab. (2018) 64:1491–500. doi: 10.7754/Clin.Lab.2018.180334, PMID: 30274009

[30] Panitchote A Mehkri O Hastings A Hanane T Demirjian S Torbic H. Clinical predictors of renal non-recovery in acute respiratory distress syndrome. BMC Nephrol. (2019) 20:255. doi: 10.1186/s12882-019-1439-2, PMID: 31291909 PMC6617675

[31] Confalonieri M Salton F Fabiano F. Acute respiratory distress syndrome. Eur Respir Rev. (2017) 26:160116. doi: 10.1183/16000617.0116-2016, PMID: 28446599 PMC9488505

