# Supplementary material for: Nomogram risk prediction model for acute respiratory distress syndrome following acute kidney injury
Source: Front Med (Lausanne). 2025 Apr 9;12:1563425. doi: 10.3389/fmed.2025.1563425 (PMC12014638; doi:10.3389/fmed.2025.1563425)
Supplement: Supplementary file 1 [file Table_1.docx]

**Supplementary Table S1**

**Table S1**. STROBE Statement—Checklist of items that should be included in reports of ***cohort studies***

|  | Item No. | Recommendation | Page No. |
| --- | --- | --- | --- |
| **Title and abstract** | 1 | (*a*) Indicate the study’s design with a commonly used term in the title or the abstract | 1 |
|  |  | (*b*) Provide in the abstract an informative and balanced summary of what was done and what was found | 2 |
| Introduction | | |  |
| Background/rationale | 2 | Explain the scientific background and rationale for the investigation being reported | 3-4 |
| Objectives | 3 | State specific objectives, including any prespecified hypotheses | 3-4 |
| Methods | | | 5-8 |
| Study design | 4 | Present key elements of study design early in the paper | 5-6 |
| Setting | 5 | Describe the setting, locations, and relevant dates, including periods of recruitment, exposure, follow-up, and data collection | 5-6 |
| Participants | 6 | (*a*) Give the eligibility criteria, and the sources and methods of selection of participants. Describe methods of follow-up | 5-7 |
|  |  | (*b*) For matched studies, give matching criteria and number of exposed and unexposed | 5-7 |
| Variables | 7 | Clearly define all outcomes, exposures, predictors, potential confounders, and effect modifiers. Give diagnostic criteria, if applicable | 7 |
| Data sources/ measurement | 8* | For each variable of interest, give sources of data and details of methods of assessment (measurement). Describe comparability of assessment methods if there is more than one group | 7 |
| Bias | 9 | Describe any efforts to address potential sources of bias | 7-8 |
| Study size | 10 | Explain how the study size was arrived at | 5-7 |
| Quantitative variables | 11 | Explain how quantitative variables were handled in the analyses. If applicable, describe which groupings were chosen and why | 7 |
| Statistical methods | 12 | (*a*) Describe all statistical methods, including those used to control for confounding | 7-8 |
|  |  | (*b*) Describe any methods used to examine subgroups and interactions | 7-8 |
|  |  | (*c*) Explain how missing data were addressed | 7-8 |
|  |  | (*d*) If applicable, explain how loss to follow-up was addressed | NA |
|  |  | (*e*) Describe any sensitivity analyses | 8 |
| Results | | | 9-11 |
| Participants | 13* | (a) Report numbers of individuals at each stage of study—eg, numbers potentially eligible, examined for eligibility, confirmed eligible, included in the study, completing follow-up, and analysed | 9 |
|  |  | (b) Give reasons for non-participation at each stage | 9 |
|  |  | (c) Consider use of a flow diagram | Figure 1 |
| Descriptive data | 14* | (a) Give characteristics of study participants (eg, demographic, clinical, social) and information on exposures and potential confounders | 9-10 |
|  |  | (b) Indicate number of participants with missing data for each variable of interest | Table S2 |
|  |  | (c) Summarise follow-up time (eg, average and total amount) | NA |
| Outcome data | 15* | Report numbers of outcome events or summary measures over time | Table 1 |
| Main results | 16 | (*a*) Give unadjusted estimates and, if applicable, confounder-adjusted estimates and their precision (eg, 95% confidence interval). Make clear which confounders were adjusted for and why they were included | 9-10,  Table 3 |
|  |  | (*b*) Report category boundaries when continuous variables were categorized | 9-10 |
|  |  | (*c*) If relevant, consider translating estimates of relative risk into absolute risk for a meaningful time period | 9-10 |
| Other analyses | 17 | Report other analyses done—eg, analyses of subgroups and interactions, and sensitivity analyses | Table 4 |
| Discussion | | | 11-13 |
| Key results | 18 | Summarise key results with reference to study objectives | 11 |
| Limitations | 19 | Discuss limitations of the study, taking into account sources of potential bias or imprecision. Discuss both direction and magnitude of any potential bias | 12-13 |
| Interpretation | 20 | Give a cautious overall interpretation of results considering objectives, limitations, multiplicity of analyses, results from similar studies, and other relevant evidence | 12-13 |
| Generalisability | 21 | Discuss the generalisability (external validity) of the study results | NA |
| Other information | | |  |
| Funding | 22 | Give the source of funding and the role of the funders for the present study and, if applicable, for the original study on which the present article is based | 14-15 |

*Give information separately for exposed and unexposed groups.

**Supplementary Table S2**

**Table S2.** Ratio of missing data for the included variables.

| Characteristics | Study cohort  (n=1012)  missing data, % | Validation cohort  (n=229)  missing data, % | Total cohort  (n=1241)  missing data, % |
| --- | --- | --- | --- |
| Sex | 0% | 0% | 0% |
| Age (years) | 0% | 0% | 0% |
| Smoking history | 0% | 0% | 0% |
| Alcohol consumption | 0% | 0% | 0% |
| Diabetes mellitus history | 0% | 0% | 0% |
| BMI (kg/m^2^) | 0% | 0% | 0% |
| ALB (g/L) | 0% | 0% | 0% |
| Cys C (mg/L) | 0.8% | 0.6% | 1.4% |
| 24-hour urine protein(g/d) | 7.9% | 6.7% | 14.6% |
| SBP/DBP/MAP (mmHg) | 0% | 0% | 0% |
| Hemoglobin (g/L) | 1.2% | 1.9% | (3.1% |
| WBC (×10^9^) | 3.7% | 2.9% | 6.6% |
| Uric acid (μmol/L) | 2.7% | 4.9% | 7.6% |
| NGAL (ng/mL) | 8.3% | 10.2% | 18.5% |
| FN (g/L) | 9.1% | 6.7% | 15.8% |
| D-dimer (μg/L) | 6.8% | 2.1% | 8.6% |
| ESR (mm/h) | 0.2% | 0.3% | 0.5% |
| LDL (mmol/L) | 0.81% | 0.6% | 1.41% |
| HDL (mmol/L) | 0.78% | 0.5% | 1.28% |
| TG (mmol/L) | 1.01% | 2.3% | 3.31% |
| ALT (U/L) | 0.4% | 0.3% | 0.7% |
| AST (U/L) | 0.4% | 0.3% | 0.7% |
| AKI stage | 2.7% | 1.8% | (4.5% |
| AKI cause/Injury factors | 6.8% | 7.9% | 14.7% |

Abbreviations: BMI, body mass index; ALB, albumin; Cys C, cystatin C; SBP, systolic blood pressure; DBP, diastolic blood pressure; MAP, mean artery pressure; WBC, white blood cell; NGAL, neutrophil gelatinase-associated lipocalin; FN, fibronectin; ESR, erythrocyte sedimentation rate; LDL, low-density lipoprotein; HDL, high-density lipoprotein; TG, triglyceride; ALT, alanine aminotransferase; AST, aspartate aminotransferase. P<0.05 was statistically significant.
